# Real-world Inter-rater Agreement of PI-QUAL Version 2 for Prostate Magnetic Resonance Imaging Quality Assessment and Its Association with Diagnostic Accuracy

**DOI:** 10.1016/j.euros.2025.12.019

**Published:** 2026-01-06

**Authors:** Daniel L. van den Kroonenberg, Jelle Barentsz, Bo J. Hamstra, Stijn M. van den Bosch, Joris-Jan Gijsbertsen, Johannes B. Reitsma, Giorgio Brembilla, Iztok Caglic, H.P.J. Raat, Maarten de Rooij, Arnoud W. Postema, Francesco Giganti, Jorg R. Oddens

**Affiliations:** a Department of Urology, Amsterdam UMC, Amsterdam, The Netherlands; b Cancer Centre Amsterdam, Amsterdam, The Netherlands; c Andros Clinics, Baarn, The Netherlands; d UMC Utrecht, Julius Center for Health Sciences and Primary Care, UMC Utrecht, Utrecht University, Utrecht, The Netherlands; e Department of Radiology, IRCCS San Raffaele Scientific Institute, Vita-Salute San Raffaele University, Milan, Italy; f Department of Radiology, Addenbrooke’s Hospital and University of Cambridge, Cambridge, UK; g Department of Radiology, University College London Hospital NHS Foundation Trust, London, UK; h Department of Radiology, Laurentius Hospital, Roermond, The Netherlands; i Department of Medical Imaging, Radboud University Medical Center, Nijmegen, The Netherlands; j Department of Urology, Leiden University Medical Center, Leiden, The Netherlands; k Division of Surgery and Interventional Science, University College London, London, UK

**Keywords:** Prostate, Magnetic resonance imaging, Prostate Imaging Quality, Image quality, Diagnostic performance, Prostate Imaging Reporting and Data System

## Abstract

**Background and objective:**

Magnetic resonance imaging (MRI) has been shown to improve the detection of prostate cancer, the second most diagnosed cancer among men. This study evaluates the inter-rater agreement for MRI quality using Prostate Imaging Quality (PI-QUAL) v2. It studies the association of PI-QUAL with diagnostic accuracy and the proportion of indeterminate Prostate Imaging Reporting and Data System (PI-RADS) 3 lesions with PI-QUAL scores.

**Methods:**

This multicenter cohort study included biopsy-naïve patients from the PCAVISION trial (NCT06281769) who underwent MRI for a suspicion of prostate cancer. Four radiologists independently scored PI-QUAL v2 and PI-RADS. PI-QUAL v2 scores were dichotomized as PI-QUAL 1 versus PI-QUAL ≥2. PI-RADS was dichotomized as PI-RADS ≤2 versus PI-RADS ≥3 and evaluated using percentage agreement, kappa, and Gwet’s agree coefficient (AC) 1. The PI-QUAL consensus score was defined by the majority score or by a referee if no majority was reached. In cases with biopsy pathology, the association between PI-QUAL and diagnostic accuracy was assessed.

**Key findings and limitations:**

In total, 352 MRI scans were included, with 150 multiparametric MRI and 202 biparametric MRI scans. The percentage agreement for dichotomized PI-QUAL was 53% (95% confidence interval [CI] 51–56%) with AC1 of 0.11 (95% CI 0.06–0.16), mainly due to one reader who strictly applied technical criteria classifying 83% as inadequate (PI-QUAL 1), compared with 12–43% by others. Exclusion of this reader resulted in a percentage agreement of 69% (95% CI 65–73%) and an AC1 of 0.51 (95% CI 0.44–0.58). Consensus classified 24% of MRI scan as PI-QUAL 1 and 76% as PI-QUAL ≥2. In an exploratory analysis, the negative predictive value was 68% (95% CI 47–85%) for PI-QUAL 1 and 74% (95% CI 60–85%) for PI-QUAL ≥2, and the positive predictive value was 62% (95% CI 45–78%) and 50% (95% CI 42–59%), respectively. The proportion of PI-RADS 3 lesions was higher in PI-QUAL 1 scans than in PI-QUAL ≥2 scans (difference of –6.5%, 95% CI –11% to –1.4%, *p* = 0.011).

**Conclusions and clinical implications:**

Strict adherence to the technical PI-QUAL criteria led to poor inter-rater agreement, while visual-based assessment yielded moderate agreement. Importantly, higher PI-QUAL scores were linked to fewer indeterminate PI-RADS 3 lesions.

**Patient summary:**

We studied how doctors agree when judging the quality of prostate magnetic resonance imaging (MRI) scan using the Prostate Imaging Quality scoring system. When they focused too strictly on technical rules, agreement was poor; when scans were judged visually, agreement improved. Better-quality MRI scans led to fewer unclear (“indeterminate”) results, helping doctors make clearer biopsy decisions.

## Take Home Message

Strict adherence to the technical rules of the Prostate Imaging Quality system reduced consistency between readers. Visual-based scoring improved agreement, and higher image quality was linked to fewer indeterminate Prostate Imaging Reporting and Data System 3 lesions.

## 1 Introduction

Prostate cancer (PCa) is the second most diagnosed cancer among men worldwide, with an estimated 1.4 million new cases annually [1]. Accurate diagnostic assessment is crucial for ensuring timely detection and treatment of clinically significant PCa (csPCa), while preventing overtreatment of indolent PCa [2], [3]. These requirements have led to a shift from systematic biopsies (SBx) to magnetic resonance imaging (MRI)-guided targeted biopsies (TBx). MRI has been shown to improve the detection of csPCa, reduce unnecessary biopsies, and minimize the overdiagnosis of indolent PCa [4], [5], [6].

The growing demand for prostate MRI has underscored the need for standardization of acquisition and reporting of prostate MRI, leading to the development of Prostate Imaging Reporting and Data System (PI-RADS) v2.1 [7]. Despite this guideline, notable variation remains in prostate MRI quality and interpretation [8], [9], [10], [11]. Variation in quality could affect diagnostic accuracy negatively and increase uncertainty in MRI-based decision-making [8], [9], [12], [13].

To address this, the Prostate Imaging Quality (PI-QUAL) scoring system was developed for standardized reporting and evaluation of MRI quality. The initial version evaluated image quality on a 5-point scale with 34 criteria and technical prerequisites [14]. First studies showed that lower PI-QUAL scores were more likely to be reported with PI-RADS 3 lesions, suggesting an association between quality and diagnostic certainty [15], [16]. Moreover, studies reported lower positive predictive values (PPVs) and reduced detection rates for csPCa in examinations with PI-QUAL v1 scores of ≤4 [15]. However, version 1 also presented several challenges, including limitations with multiparametric MRI (mpMRI), moderate inter-rater agreement, and reduced usability due to the detailed 5-point scale.

In response, PI-QUAL v2 was developed to simplify the scoring process, enhance reproducibility, and include evaluation of biparametric MRI (bpMRI) [17]. Recent studies have demonstrated moderate to very good inter-rater agreement of PI-QUAL v2 [18], [19], [20]. However, all analyses were limited by the use of mpMRI only; single-center studies designs [18], [19], and one involved consensus training before the study [20]. Moreover, none assessed the association of PI-QUAL v2 with diagnostic accuracy.

This multicenter study aims to evaluate the inter-rater agreement for PI-QUAL v2 under real-world conditions and to explore its association with diagnostic accuracy and PI-RADS. This addresses a critical evidence gap regarding the reproducibility and clinical validity of PI-QUAL v2 in routine prostate MRI practice.

## 2 Patients and methods

### 2.1 Study design and population

Assessment of PI-QUAL v2 and PI-RADS V2.1 was done within the framework of the PCAVISION trial (NCT06281769) to report on the quality of the MRI images [21]. This fully paired diagnostic trial compared two imaging-based strategies for PCa detection: artificial intelligence (AI)-assisted multiparametric ultrasound (mpUS) with TBx and MRI TBx in case of suspicious lesions. Eligibility criteria of the PCAVISION trial are described in Supplementary Table 1. In the paired analysis of the PCAVISION trial, only patients with successful complete imaging for both modalities were included and were consequently included in this assessment of MRI quality. This study adheres to the Guidelines for Reporting Reliability and Agreement Studies [22].

### 2.2 MRI acquisition

MRI scans were performed at five high-volume (500–1000 prostate MRI scans per year) Dutch medical centers. The MRI sequences required were T2-weighted imaging, diffusion-weighted imaging (DWI), and DWI-derived apparent diffusion coefficient maps. The use of dynamic contrast-enhanced (DCE) imaging was dependent on the local standard of care. Detailed information on MRI acquisition parameters is provided in Supplementary Table 2.

### 2.3 PI-QUAL v2 and readers

Four genitourinary radiologists, classified as experts according to the European Society of Urogenital Radiology (ESUR)/European Association of Urology Section of Urologic Imaging consensus and not affiliated with the participating centers in the PCAVISION trial, retrospectively assessed all eligible prostate MRI scans for image quality using PI-QUAL v2 [13], [17]. Three radiologists were part of the PI-QUAL working group of the ESUR. The MRI scans were assessed independently using an online platform (RAIQC) [23]. No consensus meeting or specific PI-QUAL training was conducted. Each MRI scan was presented with prostate-specific antigen (PSA), prostate volume, PSA density, and MRI prerequisites.

The PI-QUAL v2 scoring sheet features ten criteria regarding the identification of essential prostatic structures, technical MRI quality, and the presence of artifacts [15]. If any of the technical requirements are not met, the sequence is automatically assigned 0 points.

A consensus PI-QUAL score was determined for each MRI scan by majority score. In the absence of a majority score, a fifth radiologist acted as a referee, and the referee’s score was considered final.

### 2.4 Association of MRI quality with diagnostic accuracy

To perform an exploratory analysis of the association between the (consensus) PI-QUAL V2 score and diagnostic outcomes, we included only cases with histopathological results from the PCAVISION trial. In this trial, patients underwent both MRI and AI-assisted mpUS and independent TBx in case of suspicious lesions, and SBx were optional.

The following definitions were applied: true positive = csPCa (International Society of Urological Pathology [ISUP] ≥2) in MRI TBx with positive MRI (PI-RADS ≥3), false negative = csPCa in SBx or mpUS TBx, not found with MRI TBx, true negative = benign biopsies or ISUP 1 in SBx or mpUS TBx and negative MRI (PI-RADS ≤2), and false positive = benign or ISUP 1 TBx with positive MRI [24]. Diagnostic accuracy (patient level) was assessed in terms of sensitivity, specificity, PPV, and negative predictive value (NPV).

### 2.5 Score dichotomization

PI-QUAL and PI-RADS were dichotomized based on clinical recommendations. PI-QUAL was classified as low (score = 1) or adequate (score ≥2) quality, and PI-RADS was categorized as negative (score ≤2) or positive (score ≥3) MRI.

### 2.6 Statistical analyses

Demographic data were summarized using descriptive statistics. Continuous variables were presented as medians with interquartile ranges, and categorical variables as frequencies and percentages. Inter-rater agreement for PI-QUAL and PI-RADS was assessed using percentage agreement (PA), Fleiss’ kappa for three or more raters, Cohen’s kappa for two raters, and Gwet’s [25] agreement coefficient (AC) 1. PI-QUAL group differences were analyzed using mixed-effect logistic regression with a logit link. To account for the clustered data structure, the model included random intercepts for both the patient and the rater. The PI-QUAL group was specified as a fixed effect. No random slopes were included, as these did not improve model fit and were not supported by the number of raters. Analyses were performed using StataSE 17.

## 3 Results

### 3.1 Baseline characteristics

The present study comprised 150 mpMRI and 202 bpMRI scans (Table 1). Among the 352 patients, none underwent SBx only, 70 underwent TBx guided by AI-assisted mpUS alone, 43 underwent MRI TBx alone, and 145 underwent both AI-assisted mpUS and MRI TBx. In 41% of cases, SBx was performed in addition to TBx.

**Table 1:** Baseline characteristics

	Total (*n* = 352)
Age (yr), median (IQR)	68 (63–73)
PSA (ng/ml), median (IQR)	7.4 (5.4–10)
Prostate volume (ml), median (IQR)	50 (37–62)
MRI protocol, *n* (%)	
Multiparametric	150 (43)
Biparametric	202 (57)
3 Tesla MRI, *n* (%)	350 (99)
MRI results, *n* (%)^a^	
PI-RADS ≤2	149 (42)
PI-RADS 3	39 (11)
PI-RADS ≥4	164 (47)
MRI per center, *n* (%)	–
Center 1	48 (14)
Center 2	101 (29)
Center 3	51 (14)
Center 4	70 (20)
Center 5	82 (23)
Biopsy status and results, *n* (%)	
No biopsy	94 (27)
Benign	104 (30)
ISUP 1	38 (11)
ISUP 2	38 (11)
ISUP 3	36 (10)
ISUP 4	28 (7.3)
ISUP 5	14 (4.0)

IQR = interquartile range; ISUP = International Society of Urological Pathology; MRI = magnetic resonance imaging; PI-RADS = Prostate Imaging Reporting and Data System; PSA = prostate-specific antigen.

a Derived from the PCAVISION study.

### 3.2 Inter-rater agreement of PI-QUAL

The PA among all four radiologists was 67% (95% confidence interval [CI] 65–69%), with an AC1 of 0.04 (95% CI −0.02 to 0.10) and kappa of 0.03 (95% CI –0.02 to 0.08), for the complete PI-QUAL score, and 53% (95% CI 51–56%), with an AC1 of 0.11 (95% CI 0.06–0.16) and kappa of 0.03 (95% CI –0.02 to 0.08), for dichotomized PI-QUAL (Table 2, Table 3).

**Table 2:** Inter-rater agreement based on the complete PI-QUAL v2 score (three categories)

	Weighted percentage agreement (95% CI)^a^	Weighted Gwet’s agreement coefficient 1 (95% CI)^a^	Weighted kappa (95% CI)^a^
Radiologist 1 – radiologist 2	44 (40–49)	–0.51 (–0.63 to –0.40)	0.07 (0.05–0.09)
Radiologist 1 – radiologist 3	78 (75–82)	0.46 (0.36 to 0.55)	0.24 (0.14–0.34)
Radiologist 1 – radiologist 4	70 (66–73)	0.11 (0.00 to 0.22)	0.25 (0.19–0.30)
Radiologist 2 – radiologist 3	56 (52–61)	–0.13 (–0.26 to 0.00)	0.03 (–0.02 to 0.07)
Radiologist 3 – radiologist 4	73 (70–77)	0.23 (0.12–0.34)	0.17 (0.09–0.25)
Radiologist 4 – radiologist 2	82 (79–85)	0.63 (0.56–0.70)	0.17 (0.09–0.25)
All radiologists	67 (65–69)	0.04 (–0.02 to 0.10)	0.03 (–0.02 to 0.08)
All radiologists (minus radiologist 2)	74 (72–76)	0.24 (0.17–0.31)	0.15 (0.08–0.22)

CI = confidence interval; PI-QUAL = Prostate Imaging Quality.

a Quadratic weighting.

**Table 3:** Inter-rater agreement based on the dichotomized PI-QUAL v2 score^a^

	Percentage agreement (95% CI)	Gwet’s agreement coefficient 1 (95% CI)	Kappa (95% CI)
Radiologist 1 – radiologist 2	29 (24–33)	–0.42 (–0.52 to –0.33)	0.05 (0.03–0.73)
Radiologist 1 – radiologist 3	80 (76–84)	0.73 (0.66–0.80)	0.27 (0.14–0.39)
Radiologist 1 – radiologist 4	66 (61–71)	0.43 (0.33–0.53)	0.23 (0.15–0.31)
Radiologist 2 – radiologist 3	30 (25–35)	–0.40 (–0.50 to –0.31)	–0.01 (–0.05 to 0.04)
Radiologist 3 – radiologist 4	62 (57–67)	0.33 (0.22–0.44)	0.18 (0.09–0.27)
Radiologist 4 – radiologist 2	54 (49–59)	0.14 (0.03–0.25)	0.16 (0.09–0.22)
All radiologists	53 (51–56)	0.11 (0.06–0.16)	0.03 (–0.02 to 0.08)
All radiologists (minus radiologist 2)	69 (65–73)	0.51 (0.44–0.58)	0.18 (0.10–0.26)

CI = confidence interval; PI-QUAL = Prostate Imaging Quality.

a PI-QUAL was dichotomized into inadequate (PI-QUAL 1) and adequate (PI-QUAL ≥2) quality.

The pairwise agreements involving radiologist 2 (R2) showed significant lower levels of agreement than those without involving R2. This discrepancy arose from R2's strict adherence to the PI-QUAL v2 technical requirements, while the other readers appeared to prioritize visual assessment and diagnostic interpretability. Given this fundamental divergence, a post hoc analysis was performed without R2. Inter-rater agreement improved substantially, with the dichotomized PI-QUAL score reaching a PA of 69% (95% CI 65–73%; Table 2, Table 3). Given the different scoring methodology, R2’s scores were excluded from consensus PI-QUAL score, diagnostic performance, and PI-RADS 3 analyses [25].

After consensus, 24% of MRI scans were classified as PI-QUAL 1 and 76% as PI-QUAL ≥2.

For mpMRI, 188 examinations (31%) were scored as PI-QUAL 1 and 412 (69%) as PI-QUAL ≥2 (all radiologists). For bpMRI, 368 examinations (46%) were scored as PI-QUAL 1 and 440 (54%) as PI-QUAL ≥2 (all radiologists). For mpMRI, PA across all radiologists was 56% (95% CI 52–59%), with an AC1 of 0.22 (95% CI 0.14–0.31) and kappa of –0.12 (95% CI –0.17 to –0.07). For bpMRI, PA was 52% (95% CI 49–55%), with an AC1 of 0.04 (95% CI –0.02 to 0.11) and kappa of 0.01 (95% CI –0.05 to 0.07; Supplementary Table 4). Inter-rater agreement was also assessed per MRI sequence, T stage, and center (Supplementary Tables 5–7).

### 3.3 Diagnostic accuracy

An exploratory analysis was performed on the 258 patients with biopsy results. The NPV was 68% (95% CI 47–85%) for PI-QUAL 1 compared with 74% (95% CI 60–85%) for PI-QUAL ≥2. The PPV was 62% (95% CI 45–78%) for PI-QUAL 1 and 50% for PI-QUAL ≥2 (95% CI 42–59%). The area under the receiver operating characteristic curve (AUROC) was 0.65 (95% CI 0.53–0.76) for PI-QUAL 1 and 0.60 (95% CI 0.54–0.66) for PI-QUAL ≥2 (Table 4). Sensitivity analyses per reader and for different biopsy indications (PI-RADS ≥4 and ≥3 with PSA density ≥0.10) are presented in Supplementary Tables 8 and 9.

**Table 4:** Comparison of diagnostic accuracy between the two MRI-quality groups based on the PI-QUAL consensus score

	PI-QUAL 1 (*n* = 62)^a^	PI-QUAL ≥2 (*n* = 196)^b^
PPV (95% CI)	62 (45–78)	50 (42–59)
NPV, (95% CI)	68 (47–85)	74 (60–85)
Sensitivity (95% CI)	74 (55–88)	83 (73–90)
Specificity (95% CI)	55 (36–73)	38 (29–48)
AUROC (95% CI)	0.65 (0.53–0.76)	0.60 (0.54–0.66)
Benign (%)	40 (25/62)	40 (79/196)
ISUP 1 (%)	10 (6/62)	16 (32/196)
ISUP ≥2 (%)	50 (31/62)	43 (85/196)

AUROC = area under the receiver operating characteristic curve; CI = confidence interval; ISUP = International Society of Urological Pathology; MRI – magnetic resonance imaging; NPV = negative predictive value; PI-QUAL = Prostate Imaging Quality; PPV = positive predictive value.

a Out of 86 patients with a PI-QUAL consensus score of 1; not all patients underwent prostate biopsy as the reference standard.

b Out of 266 patients with a PI-QUAL consensus score of ≥2; not all patients underwent prostate biopsy as the reference standard.

### 3.4 Proportion of PI-RADS 3 and agreement

For the PI-RADS 3 analyses, the total number of PI-RADS assessments was 1056 (three radiologists × 352 patients). Table 5 presents the distribution of PI-RADS categories across PI-QUAL categories. The proportion of PI-RADS 3 was significantly lower when image quality was higher: 19% for PI-QUAL 1 and 13% for PI-QUAL ≥2 scans (difference of –6.5%, 95% CI –11% to –1.4%, *p* = 0.011). The PA among all four radiologists for dichotomized PI-RADS was 77% (95% CI 74–79%), with an AC1 of 0.55 (95% CI 0.49–0.61) and kappa of 0.51 (95% CI 0.45–0.57).

**Table 5:** Distribution of PI-RADS^a^ categories per PI-QUAL category (individual scores)

	PI-QUAL 1	PI-QUAL ≥2
PI-RADS ≤2, *n* (%)	110 (42)	323 (41)
PI-RADS 3, *n* (%)	50 (19)	99 (13)
PI-RADS ≥4, *n* (%)	104 (39)	370 (47)
Total	264	792

PI-QUAL = Prostate Imaging Quality; PI-RADS = Prostate Imaging Reporting and Data System.

a PI-RADS score is derived from individual readers (minus radiologist 2).

## 4 Discussion

This multicenter study was designed to evaluate the real-world reproducibility of PI-QUAL v2 and observed poor inter-rater agreement. We discovered two different interpretation methodologies: one based on strict adherence to the technical prerequisites and another based on a more pragmatic assessment of image quality and interpretability. When applied across all four readers, these approaches resulted in poor overall inter-rater agreement. However, when focusing on the readers who prioritized visual assessment, moderate agreement was achieved. Additionally, higher image quality was significantly associated with a lower proportion of indeterminate PI-RADS 3 lesions.

The inter-rater agreement for PI-QUAL v2 was poor among four radiologists, probably explained by the fact that one radiologist classified only 17% of MRI scans as of PI-QUAL ≥2 quality, compared with 57–88% by the others. Exclusion of this radiologist increased the inter-rater agreement (AC1 = 0.46), more in line with the agreement observed in other studies (Lee et al’s [18] study: kappa = 0.54; Ponsiglione et al’s [19] study: AC1 = 0.55). The discrepancy appeared to be the accurate application of the PI-QUAL v2 instructions, which mandate a score of 1 if technical prerequisites are not met. The other readers, including the members of the PI-QUAL working group, overrode this rule in favor of their visual judgment (eg, clarity of zonal anatomy, signal-to-noise ratio, and absence of motion artifacts) and not considering the technical prerequisites. This raises questions about the PI-QUAL score: are the technical prerequisites (eg, DWI slice thickness <4 mm and time resolution <15 s) too stringent, unnecessarily penalizing scans that remain diagnostically interpretable for experts? Or is visual assessment by experts so subjective that it requires the rigid guardrails of technical prerequisites? Future iterations should either prioritize visual assessment, with technical parameters serving as guidance only, or provide a more nuanced system that does not automatically assign a failing grade for a single technical deviation.

Three readers were experienced in PI-QUAL v2 and contributed to its development, while one had less experience. However, we did not observe clear differences between experienced and less experienced readers, which may be due to the simplified PI-QUAL v2 scoring or that all radiologists were expert in prostate MRI. Moreover, we did not conduct a training session, unlike Orman et al [20], who reported very good pairwise agreement (AC1 = 0.82–0.90), likely attributable to their consensus meeting. This suggests that PI-QUAL v2 is not a tool that can reliably be used “off the shelf.” Such calibration will likely be essential prior to reliable implementation in clinical practice.

We studied the inter-rater agreement of PI-QUAL v2 stratified by MRI protocol and found that the agreement was higher for mpMRI than for bpMRI. Notably, 46% of bpMRI scans were rated PI-QUAL 1, compared with 31% of mpMRI scans. This aligns with the findings of Lee et al [18], who observed a significantly higher proportion of lesions with PI-QUAL ≥2 in mpMRI scans than in bpMRI scans (64% vs 30%). These subgroup results build on the hypothesis that DCE imaging could serve as a safety net for image quality.

In contrast to PI-QUAL, the inter-rater agreement for PI-RADS was good between pairs (PA ranging from 90% to 94%) and good between all radiologists (PA of 92%). These results are consistent with those of two prior systematic reviews and meta-analyses [26], [27]. The higher inter-rater agreement observed for PI-RADS than for PI-QUAL may be explained by its broader implementation, potentially leading to greater alignment among radiologists. Our findings suggest that, despite the differing interpretations of image quality by the radiologists, they reach the same diagnostic conclusions in our dataset.

We investigated in an exploratory analysis how PI-QUAL is associated with diagnostic accuracy in patients with biopsy results. However, because biopsy indication was based on two different imaging modalities, this comparison is subject to a verification bias. Compared with PI-QUAL 1 scans, PI-QUAL ≥2 scans showed a 12% lower PPV. Clinically, this means that when imaging was interpreted as suggestive of cancer, the proportion of men who truly had cancer on biopsy was lower in PI-QUAL ≥2 scans. One likely explanation is that improvement in image quality increases the number of lesions judged suspicious, including some that ultimately prove benign, thereby reducing PPV. In contrast, PI-QUAL ≥2 scans showed a 6% higher NPV. This indicates that when imaging was interpreted as not suspicious, the probability of being truly cancer free was modestly higher in high-quality scans. This aligns with the expectation that higher-quality MRI reduces the likelihood of false negative imaging findings. Importantly, the AUROC was similar across PI-QUAL categories, suggesting that while image quality modestly shifts predictive values, it does not substantially alter the overall discriminatory ability of MRI for csPCa in this cohort.

Similar to our findings, Pötsch et al [28] reported no significant difference in AUROC values for PI-QUAL v1 scores >3 and ≤3. In contrast, Brembilla et al [15] found a significantly higher PPV for PI-QUAL v1 >3 than for PI-QUAL v1 ≤3. The discrepancy may be explained by methodological differences, such as the use of PI-QUAL v1 and involvement of only two readers in Brembilla et al’s [15] study. These results suggest that for expert readers, these two concepts are not as tightly linked as one might assume. An imperfect quality scan may still be perfectly interpretable for diagnostic purposes. Still, because two different modalities were used to determine the biopsy result, and PI-QUAL 1 numbers are low, the association between PI-QUAL and diagnostic accuracy should be interpreted with caution.

A significantly lower proportion of PI-RADS 3 lesions was observed in PI-QUAL ≥2 than in PI-QUAL 1 (–6.5%, 95% CI –11% to –1.4%; *p* = 0.011). These findings, consistent with prior studies [15], [16], suggest that higher PI-QUAL scores increase radiologists’ confidence in interpreting prostate MRI, resulting in fewer indeterminate PI-RADS 3 lesions. This is relevant, as the detection rate is very low for PI-RADS 3 lesions, compared with PI-RADS ≥4 lesions [29]. Therefore, implementing PI-QUAL in clinical practice and, consequently, rescanning MRI scans with PI-QUAL 1, may reduce the number of PI-RADS 3 lesions and, consequently, unnecessary biopsies.

Several limitations should be addressed. First, dichotomization of PI-QUAL and PI-RADS scores may have reduced the level of detail. We adopted this approach to facilitate easy translation of our results into clinical practice. Second, we excluded one radiologist from most of our consensus scoring and clinical associations. While this improved inter-rater agreement, it may have introduced a selection bias. The exclusion was deemed justified, not because the reader was an outlier, but because the reader’s literal application of the PI-QUAL v2 technical and visual criteria differed fundamentally from the more interpretative, visual assessment–based approach adopted by the other readers. The excluded results provided valuable insights, suggesting that strict adherence to technical parameters may not always be necessary. Third, although biopsy indications were determined independently by two imaging pathways, thereby avoiding an incorporation bias, the diagnostic accuracy analysis still suffers from a verification bias. Patients with negative findings on both MRI and mpUS were not biopsied, which may have led to an overestimation of diagnostic performance. However, only 27% had double-negative imaging findings, which limits the extent of the verification bias.

## 5 Conclusions

Our study observed poor inter-reader agreement for PI-QUAL v2 when used by expert radiologists in a real-world, multicenter setting. A likely explanation for this was a fundamental difference in interpretation, with one reader strictly following the tool’s technical requirements, while others prioritized practical visual assessment. When focusing on visual assessment, moderate agreement was achieved, and higher scores were linked to significantly fewer indeterminate PI-RADS 3 lesions. These findings suggest that PI-QUAL needs further refinement, clear guidelines on the roles of technical versus visual parameters, and additional training.

  ***Author contributions:*** Daniel L. van den Kroonenberg had full access to all the data in the study and takes responsibility for the integrity of the data and the accuracy of the data analysis.

  *Study concept and design*: van den Kroonenberg, Barentsz, Giganti, Oddens.

*Acquisition of data*: van den Kroonenberg, Hamstra, Gijsbertsen.

*Analysis and interpretation of data*: van den Kroonenberg, Hamstra, Gijsbertsen, Reitsma, van den Bosch, Oddens, Postema.

*Drafting of the manuscript*: van den Kroonenberg, Barentsz, Hamstra, SM, Gijsbertsen, SJ, Brembilla, Caglic, Raat, de Rooij, Postema, Giganti, Oddens.

*Critical revision of the manuscript for important intellectual content*: Oddens.

*Statistical analysis*: van den Kroonenberg, Hamstra, Gijsbertsen, Reitsma.

*Obtaining funding*: Oddens.

*Administrative, technical, or material support*: None.

*Supervision*: Barentsz, Oddens.

*Other*: None.

  ***Financial disclosures:*** Daniel L. van den Kroonenberg certifies that all conflicts of interest, including specific financial interests and relationships and affiliations relevant to the subject matter or materials discussed in the manuscript (eg, employment/affiliation, grants or funding, consultancies, honoraria, stock ownership or options, expert testimony, royalties, or patents filed, received, or pending), are the following: Arnoud W. Postema is a scientific advisor for Angiogenesis Analytics for which he receives compensation.

  ***Funding/Support and role of the sponsor:*** The study was funded by Angiogenesis Analytics, Den Bosch, in the context of the PCAVISION trial. Angiogenesis Analytics was supported by the transition program of the European innovation (#101057919 PCaVision). Giorgio Brembilla, Iztok Caglic, H.P.J. Raat, Maarten de Rooij, and Francesco Giganti were financially supported by Angiogenesis Analytics for the time spent on this project. The sponsor played a role in management of the data.

  ***Acknowledgments:*** We gratefully acknowledge the technical support provided by the team at Angiogenesis Analytics, particularly Anna Garrido-Utrilla.

## References

[1] Bergengren O., Pekala K.R., Matsoukas K. (2023). 2022 Update on prostate cancer epidemiology and risk factors—a systematic review. Eur Urol.

[2] Narayan V., Jiang S., Warlick C.A. (2017). Early stage cancer in older adults: prostate-avoiding overtreatment and undertreatment. Cancer J.

[3] Hanna B., Ranasinghe W., Lawrentschuk N. (2019). Risk stratification and avoiding overtreatment in localized prostate cancer. Curr Opin Urol.

[4] Rouvière O., Puech P., Renard-Penna R. (2019). Use of prostate systematic and targeted biopsy on the basis of multiparametric MRI in biopsy-naive patients (MRI-FIRST): a prospective, multicentre, paired diagnostic study. Lancet Oncol.

[5] Kasivisvanathan V., Rannikko A.S., Borghi M. (2018). MRI-targeted or standard biopsy for prostate-cancer diagnosis. N Engl J Med.

[6] van der Leest M., Cornel E., Israël B. (2019). Head-to-head comparison of transrectal ultrasound-guided prostate biopsy versus multiparametric prostate resonance imaging with subsequent magnetic resonance-guided biopsy in biopsy-naïve men with elevated prostate-specific antigen: a large prospective multicenter clinical study. Eur Urol.

[7] Turkbey B., Rosenkrantz A.B., Haider M.A. (2019). Prostate Imaging Reporting and Data System version 2.1: 2019 update of Prostate Imaging Reporting and Data System version 2. Eur Urol.

[8] Giganti F., Ng A., Asif A. (2023). Global variation in magnetic resonance imaging quality of the prostate. Radiology.

[9] Stabile A., Giganti F., Kasivisvanathan V. (2020). Factors influencing variability in the performance of multiparametric magnetic resonance imaging in detecting clinically significant prostate cancer: a systematic literature review. Eur Urol Oncol.

[10] Massanova M., Vere R., Robertson S. (2023). Clinical and prostate multiparametric magnetic resonance imaging findings as predictors of general and clinically significant prostate cancer risk: a retrospective single-center study. Curr Urol.

[11] Barone B., Napolitano L., Calace F.P. (2023). Reliability of multiparametric magnetic resonance imaging in patients with a previous negative biopsy: comparison with biopsy-naive patients in the detection of clinically significant prostate cancer. Diagnostics (Basel).

[12] Sackett J., Shih J.H., Reese S.E. (2021). Quality of prostate MRI: is the PI-RADS standard sufficient?. Acad Radiol.

[13] de Rooij M., Israël B., Tummers M. (2020). ESUR/ESUI consensus statements on multi-parametric MRI for the detection of clinically significant prostate cancer: quality requirements for image acquisition, interpretation and radiologists' training. Eur Radiol.

[14] Giganti F., Allen C., Emberton M., Moore C.M., Kasivisvanathan V. (2020). Prostate Imaging Quality (PI-QUAL): a new quality control scoring system for multiparametric magnetic resonance imaging of the prostate from the PRECISION trial. Eur Urol Oncol.

[15] Brembilla G., Lavalle S., Parry T. (2023). Impact of Prostate Imaging Quality (PI-QUAL) score on the detection of clinically significant prostate cancer at biopsy. Eur J Radiol.

[16] Karanasios E., Caglic I., Zawaideh J.P., Barrett T. (2022). Prostate MRI quality: clinical impact of the PI-QUAL score in prostate cancer diagnostic work-up. Br J Radiol.

[17] de Rooij M., Allen C., Twilt J.J. (2024). PI-QUAL version 2: an update of a standardised scoring system for the assessment of image quality of prostate MRI. Eur Radiol.

[18] Lee K.L., Caglic I., Liao P.H., Kessler D.A., Guo C.Y., Barrett T. (2025). PI-QUAL version 2 image quality categorisation and inter-reader agreement compared to version 1. Eur Radiol.

[19] Ponsiglione A., Cereser L., Spina E. (2024). PI-QUAL version 2: a multi-reader reproducibility study on multiparametric MRI from a tertiary referral center. Eur J Radiol.

[20] Orman M., Sirolu S., Seker M.E. (2025). Multicenter analysis for inter reader agreement of PIQUAL score v2 for basic readers in prostate MRI. Sci Rep.

[21] van den Kroonenberg D.L., Jager A., Garrido-Utrilla A. (2024). Clinical validation of multiparametric ultrasound for detecting clinically significant prostate cancer using computer-aided diagnosis: a direct comparison with the magnetic resonance imaging pathway. Eur Urol Open Sci.

[22] Kottner J., Audige L., Brorson S. (2011). Guidelines for Reporting Reliability and Agreement Studies (GRRAS) were proposed. J Clin Epidemiol.

[23] RAIQC. A flight simulator for medical image interpretation. https://www.raiqc.com/.

[24] van Leenders G., van der Kwast T.H., Grignon D.J. (2020). The 2019 International Society of Urological Pathology (ISUP) consensus conference on grading of prostatic carcinoma. Am J Surg Pathol.

[25] Gwet K.L. (2014).

[26] Wen J., Ji Y., Han J., Shen X., Qiu Y. (2022). Inter-reader agreement of the prostate imaging reporting and data system version v2.1 for detection of prostate cancer: a systematic review and meta-analysis. Front Oncol.

[27] Lee C.H., Vellayappan B., Tan C.H. (2022). Comparison of diagnostic performance and inter-reader agreement between PI-RADS v2.1 and PI-RADS v2: systematic review and meta-analysis. Br J Radiol.

[28] Pötsch N., Rainer E., Clauser P. (2022). Impact of PI-QUAL on PI-RADS and cancer yield in an MRI-TRUS fusion biopsy population. Eur J Radiol.

[29] Oerther B., Nedelcu A., Engel H. (2024). Update on PI-RADS version 2.1 diagnostic performance benchmarks for prostate MRI: systematic review and meta-analysis. Radiology.

