# Supplementary material for: Real-world Inter-rater Agreement of PI-QUAL Version 2 for Prostate Magnetic Resonance Imaging Quality Assessment and Its Association with Diagnostic Accuracy
Source: Eur Urol Open Sci. 2026 Jan 6;84:22–8. doi: 10.1016/j.euros.2025.12.019 (PMC12809076; doi:10.1016/j.euros.2025.12.019)
Supplement: Supplementary Data 1 [file mmc1.docx]

**Supplementary Table 1:** Eligibility criteria of PCAVISION trial

| **Inclusion criteria** | |
| --- | --- |
| 1. | Age ≥18 years |
| 2. | Biopsy-naïve |
| 3. | Clinical suspicion of prostate cancer |
| 4. | Scheduled for prostate Magnetic Resonance Imaging evaluation because of a suspicious digital rectal examination and/or elevated serum prostate-specific antigen |
| 5. | Signed informed consent |
| **Exclusion criteria** | |
| 1. | Active (urinary tract) infection or prostatitis |
| 2. | History of a cardiac right-to-left shunt |
| 3. | Allergic to sulfur hexafluoride or any of the other ingredients of the ultrasound contrast agent (SonoVue) |
| 4. | Current treatment with dobutamine |
| 5. | Severe pulmonary hypertension (pulmonary artery pressure >90 mm Hg), uncontrolled systemic hypertension, or respiratory distress syndrome |
| 6. | Any (further) contraindication to Magnetic Resonance Imaging or three-dimensional multiparametric ultrasound imaging |
| 7. | History of prostate surgery |
| 8. | Current 5α-reductase inhibitor use for at least 3 months |
| 9. | Incapable of understanding the language in which the patient information is given |

| **MRI center** | **Manufacturer** | **Model** | **Field strength** | **Protocol** | **T2WI Details (planes; slice; voxel)** | **DWI Details (b-value; slice; voxel)** | **DCE Details (TR; model; injection)** | **Coils** | **Bowel relaxant** |
| --- | --- | --- | --- | --- | --- | --- | --- | --- | --- |
| **1** | Siemens | VIDA | 3T | Multiparametric | Axial, coronal, sagittal; 3 mm; 0.6×0.6×3 mm | B50–1400; 3 mm; 2×2×3 mm | 3.62 ms; Dotarem; 2.5 ml/s | Spine & body array | Yes |
| **2_a_** | Philips | Ingenia Elition | 3T | Multiparametric | Axial, coronal, sagittal; 3 mm; 0.4×0.7 mm | B200–1500; 3 mm; 3×3 mm | 35.3 s; Intellispace Portal; 2 ml/s, SUB | Anterior & Posterior Coil | No |
| **3** | Siemens | VIDA | 3T | Biparametric | Axial, coronal, sagittal; 3 mm; 0.3×0.3×3 mm | B50–800 + calc B1400; 3 mm; 0.9×0.9×3 mm | – | Pelvic array | Yes |
| **4** | Philips | Ingenia | 3T | Biparametric | Axial, coronal, sagittal; 3 mm; 0.36×0.36×0.63 mm | B50–2400; 3 mm; 1 mm | – | Torso & integrated coils | No |
| **5_a_** | Siemens | Magnetom Verio | 3T | Biparametric | Axial, sagittal; 3 mm; 0.6×0.6×3 mm | B0, B1000, B2000; 5 mm; 1.9×1.4×5 mm | – | Spine & body coil | Yes |
| MRI = Magnetic Resonance Imaging; T = Tesla; T2WI = T2-Weighted Imaging; DWI = Diffusion-Weighted Imaging; DCE = Dynamic Contrast-Enhanced imaging; B = b-value; TR = temporal resolution. _a_ Not compliant with technical prerequisites of PI-QUAL V2 | | | | | | | | | |

**Supplementary Table 2:** MRI Acquisition Parameters.

**Supplementary Table 3:** Distribution PI-QUAL and PI-RADS scores per radiologist.

|  | **Radiologist 1**  No. (%) | **Radiologist 2**  No. (%) | **Radiologist 3**  No. (%) | **Radiologist 4**  No. (%) |
| --- | --- | --- | --- | --- |
| **PI-RADS** |  |  |  |  |
| **≤ 2** | 174 (49) | 125 (36) | 112 (32) | 147 (42) |
| **3** | 36 (10) | 45 (13) | 73 (21) | 40 (11) |
| **4** | 57 (16) | 106 (30) | 66 (19) | 84 (24) |
| **5** | 85 (24) | 76 (22) | 101 (29) | 81 (23) |
| **PI-QUAL** |  |  |  |  |
| **1** | 41 (12) | 292 (83) | 71 (20) | 152 (43) |
| **2** | 94 (27) | 53 (15) | 137 (39) | 160 (45) |
| **3** | 217 (62) | 7 (2) | 144 (41) | 40 (11) |

**Supplementary Table 4:** Interrater agreement PI-QUAL v2 (dichotomized) stratified for MRI protocol_a_

|  | **Percentage agreement**  (95% CI) | **Gwet’s agreement coefficient 1** (95% CI) | **Kappa** (95% CI) |
| --- | --- | --- | --- |
| **Multiparametric MRI** (n = 147) |  |  |  |
| Radiologist 1 - Radiologist 2 | 22 (15 to 29) | -0.54 (-0.68 to -0.40) | 0.02 (0 to 0.04) |
| Radiologist 1 - Radiologist 3 | 87 (82 to 93) | 0.85 (0.78 to 0.92) | -0.03 (-0.05 to -0.01) |
| Radiologist 1 - Radiologist 4 | 81 (75 to 88) | 0.75 (0.65 to 0.85) | 0.06 (-0.12 to 0.24) |
| Radiologist 2 - Radiologist 3 | 25 (18 to 32) | -0.49 (-0.63 to -0.35) | 0.01 (-0.03 to 0.04) |
| Radiologist 3 - Radiologist 4 | 79 (73 to 86) | 0.71 (0.60 to 0.82) | 0.15 (-0.08 to 0.37) |
| Radiologist 4 - Radiologist 2 | 39 (31 to 47) | -0.21 (-0.37 to -0.05) | 0.07 (0.02 to 0.12) |
| **All radiologists** | **56 (52 to 59)** | **0.22 (0.14 to 0.31)** | **-0.12 (-0.17 to -0.07)** |
| **All radiologists (minus Radiologist 2)** | **83 (78 to 87)** | **0.77 (0.70 to 0.85)** | **0.07 (-0.05 to 0.18)** |
| **Biparametric MRI** (n = 205) |  |  |  |
| Radiologist 1 - Radiologist 2 | 34 (27 to 40) | -0.33 (-0.46 to -0.19) | 0.07 (0.03 to 0.10) |
| Radiologist 1 - Radiologist 3 | 75 (69 to 81) | 0.62 (0.51 to 0.73) | 0.17 (-0.04 to 0.37) |
| Radiologist 1 - Radiologist 4 | 54 (47 to 61) | 0.14 (-0.01 to 0.29) | 0.14 (0.04 to 0.24) |
| Radiologist 2 - Radiologist 3 | 33 (26 to 40) | -0.33 (-0.46 to -0.19) | 0.02 (-0.01 to 0.06) |
| Radiologist 3 - Radiologist 4 | 50 (43 to 56) | 0.02 (-0.13 to 0.16) | 0.09 (0 to 0.18) |
| Radiologist 4 - Radiologist 2 | 65 (58 to 71) | 0.39 (0.26 to 0.53) | 0.26 (0.15 to 0.36) |
| **All radiologists** | **52 (49 to 55)** | **0.04 (-0.02 to 0.11)** | **0.01 (-0.05 to 0.07)** |
| **All radiologists (minus Radiologist 2)** | **59 (55 to 64)** | **0.27 (0.17 to 0.37)** | **0.05 (-0.04 to 0.14)** |

_a_ PI-QUAL was dichotomized into inadequate quality (PI-QUAL 1) and adequate quality (PI-QUAL ≥ 2).

**Supplementary Table 5:** Interrater agreement PI-QUAL v2 stratified for MRI sequence.

|  | **Percentage Agreement**  (95% CI) | **Gwet’s agreement coefficient 1** (95% CI) | **Kappa**  (95% CI) |
| --- | --- | --- | --- |
| **PI-QUAL T2WI (0-2 vs. 3-4)** | | |  |
| Radiologist 1 - Radiologist 2 | 33 (28 to 37) | -0.30 (-0.41 to -0.19) | 0.05 (0.03 to 0.07) |
| Radiologist 1 - Radiologist 3 | 90 (87 to 93) | 0.87 (0.85 to 0.93) | 0.13 (-0.03 to 0.3) |
| Radiologist 1 - Radiologist 4 | 73 (69 to 78) | 0.62 (0.54 to 0.70) | 0.15 (0.06 to 0.25) |
| Radiologist 2 - Radiologist 3 | 30 (25 to 35) | -0.35 (-0.46 to -0.24) | 0.02 (-0.01 to 0.04) |
| Radiologist 3 - Radiologist 4 | 72 (68 to 77) | 0.61 (0.53 to 0.70) | 0.12 (0.03 to 0.20) |
| Radiologist 4 - Radiologist 2 | 50 (45 to 55) | 0 (-0.10 to 011) | 0.17 (0.11 to 0.23) |
| **All radiologists** | **58 (55 to 61)** | **0.29 (0.23 to 0.35)** | **-0.02 (-0.07 to 0.02)** |
| **All radiologists  (minus Radiologist 2)** | **79 (75 to 82)** | **0.72 (0.67 to 0.77)** | **0.09 (0.02 to 0.17)** |
| **PI-QUAL DWI (0-2 vs. 3-4)** | | |  |
| Radiologist 1 - Radiologist 2 | 54 (48 to 59) | 0.18 (0.07 to 0.30) | 0.14 (0.09 to 0.19) |
| Radiologist 1 - Radiologist 3 | 76 (72 to 81) | 0.67 (0.59 to 0.74) | 0.21 (0.10 to 0.32) |
| Radiologist 1 - Radiologist 4 | 72 (67 to 77) | 0.58 (0.49 to 0.67) | 0.24 (0.15 to 0.33) |
| Radiologist 2 - Radiologist 3 | 48 (43 to 54) | 0 (-0.11 to 0.12) | 0.01 (-0.08 to 0.10) |
| Radiologist 3 - Radiologist 4 | 64 (58 to 69) | 0.38 (0.27 to 0.48) | 0.14 (0.04 to 0.25) |
| Radiologist 4 - Radiologist 2 | 55 (49 to 60) | 0.10 (0 to 0.21) | 0.12 (0.02 to 0.21) |
| **All radiologists** | **61 (59 to 64)** | **0.33 (0.27 to 0.39)** | **0.10 (0.04 to 0.16)** |
| **All radiologists  (minus Radiologist 2)** | **71 (67 to 74)** | **0.55 (0.48 to 0.62)** | **0.17 (0.08 to 0.25)** |
| **PI-QUAL DCE +/-** | | |  |
| Radiologist 1 - Radiologist 2 | 63 (58 to 68) | 0.47 (0.37 to 0.57) | 0.01 (-0.01 to 0.03) |
| Radiologist 1 - Radiologist 3 | 89 (85 to 92) | 0.79 (0.71 to 0.84) | 0.76 (0.68 to 0.83) |
| Radiologist 1 - Radiologist 4 | 76 (72 to 81) | 0.61 (0.53 to 0.70) | 0.43 (0.34 to 0.52) |
| Radiologist 2 - Radiologist 3 | 66 (61 to 71) | 0.52 (0.43 to 0.61) | 0.01 (-0.01 to 0.03) |
| Radiologist 3 - Radiologist 4 | 78 (73 to 82) | 0.64 (0.56 to 0.72) | 0.44 (0.35 to 0.54) |
| Radiologist 4 - Radiologist 2 | 83 (79 to 87) | 0.80 (0.75 to 0.86) | 0.03 (-0.03 to 0.08) |
| **All radiologists** | **76 (73 to 79)** | **0.63 (0.57 to 0.70)** | **0.30 (0.26 to 0.35)** |
| **All radiologists  (minus Radiologist 2)** | **81 (78 to 84)** | **0.68 (0.61 to 0.74)** | **0.54 (0.48 to 0.61)** |

T2WI = T2-weighted imaging; DWI = diffusion-weighted imaging; DCE = dynamic contrast-enhanced

**Supplementary Table 6:** Interrater agreement PI-QUAL v2 (dichotomized) stratified per MRI Center_a_

|  | **Percentage agreement**  (95% CI) | **Gwet’s agreement coefficient 1** (95% CI) | **Kappa** (95% CI) |
| --- | --- | --- | --- |
| **Center 1** (n = 48) |  |  |  |
| Radiologist 1 - Radiologist 2 | 21 (9 to 33) | -0.52 (-0.81 to -0.22) | 0 |
| Radiologist 1 - Radiologist 3 | 96 (90 to 100) | 0.96 (0.89 to 1.0) | 0 |
| Radiologist 1 - Radiologist 4 | 98 (94 to 100) | 0.98 (0.94 to 1.0) | 0 |
| Radiologist 2 - Radiologist 3 | 21 (9 to 33) | -0.54 (-0.82 to -0.26) | -0.03 (-0.12 to 0.06) |
| Radiologist 3 - Radiologist 4 | 94 (87 to 100) | 0.93 (0.85 to 1.0) | -0.03 (-0.07 to 0.02) |
| Radiologist 4 - Radiologist 2 | 23 (11 to 35) | -0.49 (-0.79 to -0.19) | 0.01 (-0.01 to 0.04) |
| **All radiologist** | **59 (53 to 65)** | **0.38 (0.25 to 0.50)** | **-0.23 (-0.28 to -0.17)** |
| **All radiologists  (minus Radiologist 2)** | **96 (91 to 100)** | **0.96 (0.91 to 1.0)** | **-0.02 (-0.05 to 0)** |
| **Center 2** (n = 101) |  |  |  |
| Radiologist 1 - Radiologist 2 | 22 (14 to 31) | -0.55 (-0.71 to -0.37) | 0.03 (0.01 to 0.06) |
| Radiologist 1 - Radiologist 3 | 83 (76 to 91) | 0.79 (0.68 to 0.90) | 0.23 (-0.02 to 0.49) |
| Radiologist 1 - Radiologist 4 | 74 (65 to 82) | 0.60 (0.45 to 0.76) | 0.27 (0.10 to 0.44) |
| Radiologist 2 - Radiologist 3 | 27 (19 to 36) | -0.45 (-0.63 to -0.27) | 0.03 (-0.02 to 0.08) |
| Radiologist 3 - Radiologist 4 | 73 (64 to 81) | 0.56 (0.40 to 0.73) | 0.29 (0.10 to 0.48) |
| Radiologist 4 - Radiologist 2 | 47 (37 to 57) | -0.02 (-0.23 to 0.19) | 0.15 (0.07 to 0.23) |
| **All radiologist** | **54 (50 to 59)** | **0.15 (0.05 to 0.25)** | **0.01 (-0.08 to 0.10)** |
| **All radiologists  (minus Radiologist 2)** | **76 (70 to 83)** | **0.66 (0.54 to 0.77)** | **0.24 (0.09 to 0.40)** |
| **Center 3** (n = 51) |  |  |  |
| Radiologist 1 - Radiologist 2 | 45 (30 to 59) | -0.04 (-0.36 to 0.28) | 0.11 (0.01 to 0.22) |
| Radiologist 1 - Radiologist 3 | 80 (68 to 91) | 0.72 (0.53 to 0.91) | 0.27 (-0.06 to 0.60) |
| Radiologist 1 - Radiologist 4 | 57 (43 to 72) | 0.26 (-0.04 to 0.57) | 0.13 (-0.05 to 0.31) |
| Radiologist 2 - Radiologist 3 | 41 (27 to 55) | -0.17 (-0.46 to 0.13) | -0.01 (-0.21 to 0.19) |
| Radiologist 3 - Radiologist 4 | 61 (47 to 75) | 0.28 (-0.01 to 0.58) | 0.22 (-0.03 to 0.46) |
| Radiologist 4 - Radiologist 2 | 71 (58 to 85) | 0.44 (0.18 to 0.70) | 0.43 (0.19 to 0.68) |
| **All radiologist** | **59 (51 to 67)** | **0.24 (0.05 to 0.42)** | **0.12 (-0.04 to 0.28)** |
| **All radiologists  (minus Radiologist 2)** | **66 (56 to 76)** | **0.44 (0.23 to 0.64)** | **0.14 (-0.08 to 0.36)** |
| **Center 4** (n = 70) |  |  |  |
| Radiologist 1 - Radiologist 2 | 29 (17 to 39) | -0.41 (-0.65 to -0.23) | 0.05 (0 to 0.09) |
| Radiologist 1 - Radiologist 3 | 64 (53 to 76) | 0.39 (0.15 to 0.63) | 0.17 (-0.05 to 0.40) |
| Radiologist 1 - Radiologist 4 | 47 (35 to 59) | -0.05 (-0.29 to 0.20) | 0.15 (0.03 to 0.27) |
| Radiologist 2 - Radiologist 3 | 33 (22 to 44) | -0.23 (-0.51 to 0.05) | -0.13 (-0.26 to 0) |
| Radiologist 3 - Radiologist 4 | 46 (34 to 58) | -0.08 (-0.32 to 0.17) | 0.01 (-0.19 to 0.20) |
| Radiologist 4 - Radiologist 2 | 70 (59 to 81) | 0.56 (0.36 to 0.77) | 0.10 (-0.11 to 0.31) |
| **All radiologist** | **36 (30 to 42)** | **0.09 (-0.01 to 0.20)** | **-0.05 (-0.13 to 0.03)** |
| **All radiologists  (minus Radiologist 2)** | **52 (45 to 59)** | **0.07 (-0.09 to 0.22)** | **0.03 (-0.12 to 0.17)** |
| **Center 5** (n = 82) |  |  |  |
| Radiologist 1 - Radiologist 2 | 31 (21 to 42) | -0.37 (-0.58 to -0.17) | 0.07 (0.02 to 0.12) |
| Radiologist 1 - Radiologist 3 | 81 (72 to 89) | 0.73 (0.59 to 0.87) | 0.31 (0.05 to 0.58) |
| Radiologist 1 - Radiologist 4 | 57 (47 to 69) | 0.23 (0 to 0.46) | 0.19 (0.04 to 0.34) |
| Radiologist 2 - Radiologist 3 | 29 (19 to 39) | -0.42 (0.62 to -0.22) | 0.01 (-0.07 to 0.10) |
| Radiologist 3 - Radiologist 4 | 46 (35 to 57) | 0 (-0.25 to 0.25) | -0.04 (-0.21 to 0.12) |
| Radiologist 4 - Radiologist 2 | 57 (46 to 68) | 0.24 (0 to 0.47) | 0.10 (-0.07 to 0.26) |
| **All radiologist** | **50 (46 to 55)** | **0.02 (-0.07 to 0.12)** | **-0.02 (-0.11 to 0.08)** |
| **All radiologists  (minus Radiologist 2)** | **61 (54 to 69)** | **0.35 (0.19 to 0.50)** | **0.06 (-0.1 to 0.22)** |
| _a_ PI-QUAL was dichotomized into inadequate quality (PI-QUAL 1) and adequate quality (PI-QUAL ≥ 2). | | | |

**Supplementary Table 7:** Interrater agreement T-stage

|  | **Percentage agreement**  (95% CI) | **Gwet’s agreement coefficient 1** (95% CI) |
| --- | --- | --- |
| **PIQUAL 1**  **N = 60** | 0.89 (0.76 to 1) | 0.82 (0.66 to 0.99) |
| **PIQUAL ≥ 2**  **N = 194** | 0.84 (0.76 to 0.93) | 0.76 (0.66 to 0.86) |
| T-stage agreement (T2 vs T3) (three readers) | | |

**Supplementary Table 8:** Comparison of diagnostic accuracy between the two MRI-quality groups based on the PI-QUAL consensus score stratified per reader.

|  |  | **PIQUAL 1** | **PIQUAL ≥ 2** |
| --- | --- | --- | --- |
| **R1** | **PPV,** (95% CI) | 71 (44 – 90) | 67 (58 – 74) |
|  | **NPV,** (95% CI) | 50 (16 – 84) | 94 (86 – 98) |
|  | **Sensitivity,** (95% CI) | 75 (48 – 93) | 94 (87 – 98) |
|  | **Specificity,** (95% CI) | 44 (14 – 79) | 65 (56 – 73) |
|  | **AUROC,** (95% CI) | 0.60 (0.39 – 0.80) | 0.79 (0.75 – 0.84) |
| **R2** | **PPV,** (95% CI) | 62 (54 – 70) | 43 (26 – 61) |
|  | **NPV,** (95% CI) | 94 (85 – 99) | 100 (72 – 100) |
|  | **Sensitivity,** (95% CI) | 97 (92 – 99) | 100 (78 – 100) |
|  | **Specificity,** (95% CI) | 46 (36 – 56) | 36 (19 – 55) |
|  | **AUROC,** (95% CI) | 0.71 (0.67 – 0.76) | 0.68 (0.59 – 0.76) |
| **R3** | **PPV,** (95% CI) | 60 (42 – 75) | 56 (48 – 64) |
|  | **NPV,** (95% CI) | 92 (62 – 100) | 94 (83 – 99) |
|  | **Sensitivity,** (95% CI) | 96 (78 – 100) | 97 (91 – 99) |
|  | **Specificity,** (95% CI) | 42 (23 – 63) | 40 (31 – 49) |
|  | **AUROC,** (95% CI) | 0.69 (0.58 – 0.80) | 0.68 (0.63 – 0.73) |
| **R4** | **PPV,** (95% CI) | 54 (42 – 66) | 66 (57 – 75) |
|  | **NPV,** (95% CI) | 97 (85 – 100) | 95 (83 – 99) |
|  | **Sensitivity,** (95% CI) | 98 (87 – 100) | 97 (91 -100) |
|  | **Specificity,** (95% CI) | 50 (38 – 62) | 50 (38 - 62) |
|  | **AUROC,** (95% CI) | 0.74 (0.67 – 0.80) | 0.74 (0.68 – 0.80) |

PPV = positive predictive value; NPV = negative predictive value; AUROC: Area Under the Receiver Operating Characteristic; ISUP = International Society of Urological Pathology

**Supplementary Table 9:** Comparison of diagnostic accuracy between the two MRI-quality groups based on the PI-QUAL consensus score stratified for different indications of prostate biopsy.

|  |  | **PI-QUAL 1**  (n = 62)_a_ | **PI-QUAL ≥ 2** (n = 196) _b_ |
| --- | --- | --- | --- |
| **PI-RADS ≥3 + PSAD ≥0.10** | **PPV,** (95% CI) | 64 (46 to 79) | 52 (43 to 61) |
|  | **NPV,** (95% CI) | 69 (48 to 86) | 76 (63 to 86) |
|  | **Sensitivity,** (95% CI) | 74 (55 to 88) | 82 (73 to 90) |
|  | **Specificity,** (95% CI) | 58 (39 to 76) | 42 (33 to 52) |
|  | **ROC AUC** | 0.66 (0.54 to 0.78) | 0.62 (0.56 to 0.69) |
| **PI-RADS ≥4** | **PPV,** (95% CI) | 69 (52 to 83) | 62 (53 to 71) |
|  | **NPV,** (95% CI) | 83 (61 to 95) | 89 (79 to 95) |
|  | **Sensitivity,** (95% CI) | 87 (70 to 96) | 91 (82 to 96) |
|  | **Specificity,** (95% CI) | 61 (42 to 78) | 58 (48 to 67) |
|  | **ROC AUC** | 0.74 (0.64 to 0.85) | 0.74 (0.69 to 0.80) |

PPV = positive predictive value; NPV = negative predictive value; AUROC: Area Under the Receiver Operating Characteristic; ISUP = International Society of Urological Pathology

_a_Out of 86 patients with PI-QUAL consensus score of 1; not all patients underwent prostate biopsy as the reference standard

**_b_**Out of 266 patients with PI-QUAL consensus score of 2 or higher; not all patients underwent prostate biopsy as the reference standard
